# Identifying Opportunity for Online Education to Address HIV/HCV Knowledge Gaps in Health Professionals and Students in Egypt

**DOI:** 10.29024/aogh.2388

**Published:** 2018-11-05

**Authors:** Aya Khalil, Mahmoud Shaltout, Sergio A. Costa

**Affiliations:** 1 Department of Physical Therapy for Biomechanics, Cairo University, Cairo, EG; 2 Core Department, American University of Cairo, Cairo, EG; 3 Center for Systems and Community Design, City University of New York, Graduate School of Public Health and Health Policy, New York, US

## Abstract

HCV prevalence rates in Egypt are high. While low, HIV prevalence rates may be underreported. Adequate solutions to address these public health challenges are lacking. Stigma, misperceptions, and lack of specialized skills to deal with infection are persistent impediments. To address these challenges, we propose a plan that incorporates the use of online education and hybrid formats to meet the needs in the field. Online education has been used with success elsewhere in the Mediterranean and Middle East. Given the difficult economic conditions in Egypt and physical challenges of traditional formats of education, we propose a plan, curricular elements, and education tracking process as a strategy to improve the knowledge capacity of health professionals and students.

Egypt is the most populous Middle Eastern country, with a population of 95,688,680 [1]. In the midst of economic and political instability, Egypt faces numerous public health challenges, including major communicable diseases. Hepatitis C (HCV) is the country’s biggest health challenge; Egypt has the highest prevalence rate of HCV (15–20%) in the world [2,3]. Despite efficacy of recent HCV treatment options, high treatment cost and absence of infection control practices prevail [3]. Reported prevalence of Human Immunodeficiency Virus (HIV) is low in Egypt [4], but numbers may be underreported due to late diagnosis (at immunosuppression) [5] and high stigmatization [6,7,8,9], which pose a double threat.

Research on healthcare providers in Egypt show that a high number of HCV infections occur during direct patient care [10], especially during early stages of training [11]. Very little HCV infection control is applied within medical and dental care procedures [12,13,14]. Additionally, Egyptian healthcare workers exhibit unease interacting with HIV patients (touching clothes, dressing wounds, and obtaining blood samples); receive no guidelines/protocol on dealing with HIV patients [6]; and do not believe in effective control measures against HIV [9]. Stigmatization and negative attitudes (shaming, blaming) towards HIV patients by healthcare workers [6,7,8,9] further exacerbate this situation and may lead to higher incidence rates. A need for education interventions in healthcare settings has been identified with respect to both diseases [9,12].

The Egyptian Ministry of Health implemented an HCV education campaign in 2008 [14], yet infection rates remain high (26% in the Nile Delta and 28% in upper Egypt) [3]. Comparable local campaigns for HIV awareness and prevention do not exist. From a young age, Egyptians receive little reproductive health education [15] to protect them from sexually transmitted infections such as HIV. These findings highlight an urgent need to prepare a generation of public health professionals to address the challenge of stigma, misperception, and lack of HIV/HCV prevention knowledge in the population. One way to address this is for appropriate ministries in collaboration with public and private universities to invest in effective, low-cost, and easily accessible online education solutions to prepare and instruct a) healthcare workers and b) young adults/university students. These campaigns should ideally address both knowledge to improve compliance in prevention strategies/protocols and changing perceptions and attitudes towards patients.

Online education has been used in the Middle East/North Africa (MENA) region and in low- and middle-income countries [16,17,18]. As with face-to-face educational delivery models, online education is delivered in different modalities including synchronous versus asynchronous (learning in real time or accessing materials at the convenience of the student); blended learning (mixing online modules with face to face learning); and fully online (all activities and learning take place online although students could work in virtual or face-to-face groups as well as independently in the field). Modalities are selected based on preference and the appropriateness of the instructional materials to maximize positive learning outcomes. Resources are required to ensure that students have access to well-developed instructional materials and both learning and technical support. Students are likely to learn in online environments when trust in the learning management system (LMS), effective design of instructional modules, and opportunities for interactivity are present [19].

Learning institutions in many areas of the world often face budgetary and infrastructural obstacles that are exacerbated by punctuated political instabilities and persistent economic downturns such as in Egypt. These factors prevent the implementation of quality education, including online education [20]. To deliver quality online education, institutions need a stable LMS or comparable virtual space flexible enough to house materials and resources as well as create virtual space for students and instructors to interact. Using mobile devices for education allows for greater flexibility and has shown promise in Egypt [21]. In addition, access to instructional design expertise ensures that modules are consistent with cognitive learning and design principles [22,23] and access to technical and learning support to provide remedies when challenges emerge are critical factors in assuring positive learning outcomes. Traditional lectures alone are not enough to ensure mastery of learning. Key to the success of any learning program charged with teaching skills to practice professionals is creating activities for students to practice and apply what they learn.

Given the immediate urgency of both the HIV and HCV public health challenges and the logistical limitations in Egypt for reasons we stated earlier, the proposed course of action in Egypt should consist of an online, asynchronous, or blended modality certification program. The Egyptian context would potentially benefit from a fully online format, which has shown success in skill acquisition and satisfaction according to a recent systematic review [24]. Additionally, an e-learning course on reproductive health (part of a ‘Personal Health’ module) offered by the Faculty of Medicine in Cairo University showed significantly better academic results in students compared to traditional methods of instruction [25]. The asynchronous approach would allow for on-demand access to learning modules that students and stakeholders could access at their convenience. Depending on the preference of the learning coordinators, the blended format could allow for some in-person contact so that students could demonstrate knowledge and instructors could confirm mastery of skills and concepts. The certification program should consist of two distinct curricula: 1) a general course for university students preparing for a career in public or community health and 2) an advanced course for healthcare workers. Both the Ministry of Health and Population and the Ministry of Education should consider making the former a requirement for all students to graduate (as part of a ‘Personal Health’ module applicable across all universities) and a requirement of relevant healthcare professionals to continue practice with afflicted patients.

In Table 1, we propose a curriculum for university students and medical professionals. The common elements for both audiences include: HIV and HCV symptoms and transmission routes; safety tips and practices to avoid infection; addressing and correcting common public misperceptions of both illnesses; treatment and management options; and living and dealing with infected individuals. The advanced course should incorporate all of the above and include the identification of occupational health exposures of both diseases; medical safety procedures to prevent occupational exposure; use of devices with safety features; safe disposal of contaminated materials; and sensitivity training information to support stigmatized populations. The Ministry of Health should play a significant role in the provision of up-to-date information including procedures and protocols (adopting approaches used in other countries), access to equipment and infrastructure within healthcare facilities in order to implement procedures discussed in the module, and more. These courses should be made available in both English and Arabic.

**Table 1:** HIV/HCV Curriculum for University Students and Medical Professionals.

Course Topics		

	General Course (University Students)	Advanced Course (Medical Professionals)

Common Elements	An introduction to HIV and HCV: disease, symptoms, transmission routes Safety tips and practices to avoid HIV/HCV infection Public misperceptions of HIV/HCV Treatment and management options Living and dealing with infected individuals	

Different Elements		Occupational health exposures of HIV/HCV Medical safety procedures Protocols involving HIV/HCV patients Use of devices with safety features Safe disposal of contaminated materials Sensitivity training information to support stigmatized populations

We propose the following plan as a way forward. A two-year plan would be initiated by the Ministries of Health and Higher Education. Protocols would be drafted with respect to HIV/HCV treatment and implemented upon completion of the two years. A joint team with members of both ministries would be created to oversee the adoption of similar courses to those outlined above, and perhaps modify or create an equivalent for the Egyptian setting. Centers for learning and teaching within Egyptian higher learning institutions may be enlisted to help develop such courses. This will require a budget but, as mentioned previously, budgetary needs of providing traditional instruction for all universities will be offset as a result. The plan should be piloted and tested for a significant improvement in results, and the collaborative team would aim to have both modified working courses within two academic years. Throughout the two years of development, the Ministry of Health should strive to update its current health facilities or allocate resources to build several local centers serving various delta and rural communities/governorates, as centers within Cairo are already well-funded and developed. Construction of new centers may face financial constraints. A solution could include shifting resources from modifying existing centers to developing centers with basic facilities needed to reduce infection.

Certificates would be issued upon successful completion of assessments. Certification should be used as prerequisites for graduation (with respect to the general curriculum) and working/interacting with diseased individuals (with respect to the advanced continuing education) should be planned every few years. The status of re-certification should be relayed to administration of healthcare facilities to allow/restrict their doctors from dealing with HCV/HIV patients as the institutions deem appropriate as these critical needs are addressed in Egypt.
